# Deletion of SIRPα (signal regulatory protein-α) promotes phagocytic clearance of myelin debris in Wallerian degeneration, axon regeneration, and recovery from nerve injury

**DOI:** 10.1186/s12974-019-1679-x

**Published:** 2019-12-28

**Authors:** Gerard Elberg, Sigal Liraz-Zaltsman, Fanny Reichert, Takashi Matozaki, Michael Tal, Shlomo Rotshenker

**Affiliations:** 1 Medical Neurobiology, IMRIC, Faculty of Medicine, Hebrew University of Jerusalem, Ein-Kerem Campus, POB 12272, 91120 Jerusalem, Israel; 2 The Joseph Sagol Neuroscience Center, Sheba Medical Center, Kiryat Ono, Israel; 3 The Faculty of health profession, Ono Academic College, Kiryat Ono, Israel; 4 The Institute for Drug Research, Hebrew University, Jerusalem, Israel; 5 Division of Molecular and Cellular Signaling, Biochemistry and Molecular Biology, Kobe University Graduate School of Medicine, Kobe, Japan; 6 Medical Neurobiology, Faculties of Medicine and Dentistry, Center for Research on Pain, Hebrew University, Jerusalem, Israel

**Keywords:** SIRPα, Peripheral nerve, Nerve injury, Wallerian degeneration, Macrophages, Phagocytosis, Myelin, Axon regeneration

## Abstract

**Background:**

Recovery of function from traumatic nerve injury depends on the ability of severed axons to grow/regenerate back to their target tissues. This is achieved by successfully crossing the lesion site where physical impact severed axons, determined by the type of trauma, followed by successfully growing throughout the Wallerian degenerating nerve segment located distal to and beyond the lesion site, determined by the nature of Wallerian degeneration. The protracted removal of myelin debris in Wallerian degeneration, which leads residual myelin debris to slow down axon growth, impedes recovery of function. We focused in this study on mechanism(s) that delay the removal of myelin debris in Wallerian degeneration and so impede recovery. Previously, we showed that myelin debris inhibited its own phagocytosis in primary cultured macrophages and microglia as CD47 on myelin ligated SIRPα (signal regulatory protein-α) on phagocytes, and sequentially, SIRPα generated “don’t eat me” signaling. We also demonstrated that serum inhibited phagocytosis in a SIRPα-dependent manner. Herein, we aimed to determine whether SIRPα-dependent inhibition of phagocytosis in macrophages impedes the in vivo removal of myelin debris in Wallerian degeneration, further leading to impaired healing.

**Methods:**

Using SIRPα null (SIRPα−/−) and littermate wild-type (SIRPα+/+) mice, we studied the recovery of sensory and motor functions from nerve injury and, further, axon regeneration, SIRPα expression, myelin debris removal, and the phagocytic capacity and presence of macrophages in Wallerian degeneration.

**Results:**

Myelin debris removal, axon regeneration, and the recovery of functions were all faster in SIRPα−/− mice than in wild-type mice. Between the two cell types that mostly scavenge myelin debris, macrophages but not Schwann cells expressed SIRPα in wild-type mice, and furthermore, SIRPα−/− macrophages phagocytosed significantly more than wild-type macrophages.

**Conclusions:**

Our findings suggest an intrinsic normally occurring SIRPα-dependent mechanism that impedes the in vivo removal of myelin debris in Wallerian degeneration by inhibiting the phagocytosis of myelin debris in macrophages, hence preventing fast growing axons from fully implementing their regenerative potential. Thus, accelerating the removal of myelin debris by eliminating SIRPα-dependent inhibition of phagocytosis will most likely advance recovery of functions from nerve injury.

## Background

Injury to peripheral nerves produces abrupt tissue damage at the lesion site where physical impact occurs. The type of trauma determines the extent of damage. For example, compression leads to nerve crush that severs axons but largely preserves the continuity of the nerve connective tissue structures whereas cut and avulsion form a gap between proximal and distal nerve stumps. The nerve segment located distal to and beyond the lesion site undergoes Wallerian degeneration though it does not encounter the physical trauma directly [1]. Among features that characterize Wallerian degeneration and directly relate to our study, axons disintegrate, myelin sheaths that surround axons break down, a more sustained presence of numerous macrophages replaces an initial short-lived presence of fewer neutrophils, and mostly macrophages and Schwann cells scavenge myelin debris, reviewed in [2, 3].

To regain function, severed axons must reach the denervated target cells by crossing the lesion site and then entering and growing throughout the Wallerian degenerating nerve segment. Though possible, less than 50% of patients regain adequate sensory and motor functions [4, 5]. Thus, successful recovery of functions from nerve injury remains an unresolved clinical issue.

Two major factors that affect functional recovery are the type of trauma and the nature of Wallerian degeneration. The type of the trauma determines if and how many of the severed axons successfully cross the lesion site. For example, nerve crush favors successful crossing since the nerve’s uninterrupted connective tissue structures serve as conduits. In contrast, the gap between proximal and distal nerve stumps formed by nerve cut and avulsion injuries obstruct crossing, discussed in detail regarding human nerve injury [4, 5].

Wallerian degeneration follows all types of nerve injury. Our current study relates to the question how does Wallerian degeneration affect the growth of those severed axons that successfully crossed the lesion site and thereby the recovery of function. In this context, at least three factors together determine whether severed axons successfully grow/regenerate throughout the Wallerian degenerating nerve segment. First is the length of the distal nerve segment. It can vary from several millimeters to up to over 1 m depending on species (e.g., mice versus humans) and site of trauma (e.g., near to versus distant from denervated target cells). Second is the slow growth of axons, which prolongs the time it takes axons to regenerate long distances. Third is the decline with time of the capacity to support axon growth that initially develops in Wallerian degeneration. Observations in humans and studies in experimental animals support this understanding. In humans, the rate that severed axons regenerated through Wallerian degenerating nerve segments was examined at different times after nerve crush injuries that did not require surgical reconstruction and after more extensive nerve injuries that required suturing of the proximal and distal nerve stumps. Human sensory axons that regenerated at an initial rate of 2.5 mm/day slowed down to 0.5 mm/day over a period of 200 days and human motor axons that initially regenerated 2 mm/day slowed down to 1 mm/day [6, 7]. In nonhuman primates, fast regeneration was the major determinant of successful recovery [8]. In rats, axon growth support, which initially developed in Wallerian degeneration, diminished with time, resulting in poor recovery [9, 10].

A leading cause of slow axon regeneration in the Wallerian degenerating nerve segment is myelin debris (also referred to as degenerated myelin in the literature). Myelin debris and MAG (myelin-associated glycoprotein) inhibited axon growth in culture and in vivo [11–14]. Schwann cells and macrophages could alleviate axon growth inhibition since the two scavenge myelin debris in Wallerian degeneration. Schwann cells removed myelin debris by autophagy [15, 16] and phagocytosis [16, 17], and macrophages by phagocytosis [18–20]. However, the rate of myelin debris removal is very likely slower than the rate fast regenerating axons grow. This is suggested by live imaging observations that revealed the presence of residual myelin debris (i.e., myelin debris that was not yet removed) confronting and slowing down the growth of regenerating axons [21].

Myelin debris itself and serum can impede the removal of myelin debris in Wallerian degeneration. Previously, we showed that myelin debris inhibited its own phagocytosis in cultured primary macrophages and microglia through the binding of CD47 on myelin to the immune inhibitory receptor SIRPα (also known as CD172a, SHPS-1, p84, gp93, and BIT) on macrophages and microglia [22, 23]. In turn, SIRPα generated “don’t eat me” signaling, which reduced phagocytosis. We further showed that serum triggered SIRPα-dependent inhibition of myelin debris phagocytosis. As serum components are present in tissues other than central nervous system [24], serum may also exist in peripheral nerves. Therefore, not only CD47 on myelin but also serum may trigger SIRPα-dependent inhibition of myelin debris removal.

Yet, it remained unclear whether SIRPα-dependent inhibition of myelin debris phagocytosis impedes the removal of myelin debris in vivo, leading residual myelin debris to slow down axon regeneration and so delay the recovery of functions. To resolve this issue, our present study used SIRPα null (SIRPα−/−) mice and littermate wild-type (SIRPα+/+) mice [25] to analyze myelin debris removal, axon regeneration, macrophage presence and phagocytic capacity, and the recovery of sensory and motor functions. Our findings suggest that SIRPα-dependent inhibition of myelin debris phagocytosis in macrophages impedes the removal of myelin debris in Wallerian degeneration, leading to slow axon regeneration and so to delayed recovery of functions from peripheral nerve injury.

## Methods

### Animals

C57BL/6 J mice from which SIRPα was deleted (SIRPα null; homozygous SIRPα−/−) and heterozygous SIRPα+/− were generated at Kobe University Graduate School of Medicine, Japan [25]. Heterozygous SIRPα+/− were used to establish SIRPα−/− and littermate wild-type/SIRPα+/+ C57BL/6 J mice colonies at the Hebrew University Faculty of Medicine animal facility. Sex- and age-matched 8- to 12-week-old mice were used in experiments in accordance with the Israeli national research council guide for the care and use of laboratory animals and the approval of the Hebrew University institutional ethic committee.

### Surgical procedures

Surgery was performed under anesthesia on one hind limb of wild-type and SIRPα−/− mice. Sciatic and saphenous nerves were exposed through small incisions in the overlaying skin. Freeze-crush injuries that enable axon regeneration were performed on sciatic and saphenous nerves using a fine jeweler’s tweezer that was cooled in liquid nitrogen and then applied to nerves for 5 s, taking care to preserve the continuity of the epineurium. Avulsion injuries that do not enable axon regeneration were performed on sciatic nerves by removing a small nerve segment at mid-thigh level. Finally, the skin was sutured and sprayed with antiseptics.

### Assessment of functional recovery after nerve injury

Two investigators assessed the recovery of sensory and motor functions independently by testing all wild-type and SIRPα−/− mice side by side at 1-day intervals after surgery. Each mouse was tested for at least 3 days after function first returned to verify consistency.

*Recovery of sensory function* was assessed using the flexion-withdrawal reflex: withdrawal of hind limbs in response to touching their paws with a blunt pin and von-Frey monofilaments that produce punctate mechanical stimuli delivered mostly by Aδ axons, i.e., pinprick testing [26]. Mice that had their saphenous nerve freeze-crushed were placed on an elevated wire mesh platform until calm, and then, testing of both injured and uninjured limbs was carried out by gently touching paws at areas that saphenous sensory axons normally innervate.

*Recovery of motor function* was assessed using the toe-spreading reflex: spreading of the toes in response to gently lifting mice by their tail. The reflex was tested in both the injured and uninjured hind limbs.

### Preparation of BMDM (bone marrow-derived macrophage)

We followed previously published protocols [27–29] with some modifications. Femur and tibia bones were removed from wild-type and SIRP−/− mice and placed in complete MEM supplemented with 15% heated inactivated FCS, 2 mM glutamine, MEM non-essential amino acids, MEM vitamin solutions, 1 mM sodium pyruvate, 1ug/ml transferrin APO, 100 U/ml penicillin, and 100 mg/ml streptomycin (Biological Industries, Beit Haemek, Israel). Bone marrow was flushed out, cells suspended in red blood cell lysis buffer for 1 min, washed in complete MEM, and plated in cell culture petri dishes for 2 to 4 h at 37 °C. Non-adherent bone marrow-derived cells that include macrophage precursor cells were plated in 100 mm plastic/bacteriological dishes (0.4 10^6^ cells/dish) in complete MEM supplemented with 15% L929-cell conditioned medium that contains the macrophage MCSF (colony-stimulating factors) [30]. Macrophage precursor cells that differentiated into adherent BMDM after one week in the presence L929 cells conditioned media were used in experiments.

### Myelin isolation

The detailed protocol for isolating myelin was previously described [31]. Isolated myelin is “myelin debris” since intact myelin breaks down during isolation.

### Phagocytosis of myelin debris

Phagocytosis was assayed as previously described [31]. BMDM were plated in 96-well tissue culture plates at a density that minimizes cell-cell contact in the presence of DMEM supplemented by 10% FCS. Non-adherent BMDM were washed out after 2 h and adherent BMDM left to rest overnight. Next, BMDM were washed and myelin debris added in DMEM/F12 in the presence of serum for 40 min, unphagocytosed myelin debris washed out, and levels of phagocytosis determined by ELISA. At this time all myelin debris was phagocytosed/internalized [31, 32].

### Detecting and quantifying myelin debris phagocytosis by ELISA

This assay is based on the detection of the myelin-specific protein MBP (myelin basic protein) in phagocyte as previously detailed [31]. Since MBP is unique to myelin and BMDM do not produce it, MBP levels in BMDM cytoplasm are proportional to levels of phagocytosed myelin debris. In brief, BMDM were immediately lysed (50 mM carbonate buffer, pH 10) after myelin debris phagocytosis was completed, and lysates transferred to high protein absorbance plates (Thermo Fisher Scientific, Nunc International, USA) in equal volume of coating buffer (0.5 M carbonate buffer pH 9.6). Levels of MBP were determined by ELISA using rat anti-MBP mAb and matching control IgG (Bio-Rad Laboratories Inc., Hercules, USA).

When phagocytosis by BMDM from wild-type mice was compared with phagocytosis by BMDM from SIRPα−/− mice, phagocytosis by each population was first normalized to the number of respective BMDM counted in 1-mm^2^ areas at the center of wells. Normalizing phagocytosis to cell number is required since BMDM from the two strains of mice may differ in their adherence properties, thus resulting in different number of adherent cells even when the same number of cells was initially seeded. To this end, BMDM in replicate plates were fixed, stained, and counted. Phagocytosis by BMDM from SIRPα−/− mice was calculated as percentage of phagocytosis by BMDM from wild-type mice normalized to 100%.

### Quantifying MBP content in nerve tissue

The detailed protocol used to quantify Galectin-3/MAC-2 [27] was previously adopted to quantify MBP in peripheral nerves [33]. In brief, nerves were homogenized in 50 mM sodium carbonate buffer pH 9.0 supplemented with protease inhibitor cocktail (Sigma-Aldrich, Saint Louis, USA), and protein concentration in cleared extracts was determined using the Bradford assay reagent (Bio-Rad Laboratories Inc., Hercules, USA) and adjusted to 5 μg/mL. Equal volumes (75 μL) of extracts and coating buffer (0.5 M carbonate buffer pH 9.6) were incubated overnight at 4 °C in 96-well high protein absorbance plates (Thermo Fisher Scientific, Nunc International, USA), and levels of MBP determined by ELISA using rat anti-mouse MBP mAb (Bio-Rad Laboratories Inc., Hercules, USA).

### Immunoblot analysis

BMDM were plated in 10 cm tissue culture plates at a non-confluent density (3 × 10^6^ cells per plate) in the presence of DMEM supplemented by 10% FCS and left to rest overnight. BMDM were washed in PBS and lysed in ice cold lysis buffer (50 mM Tris HCL pH 7.5, 20 nM MgCl_2_, 150 mM NaCl, 0.5% NP-40, 10 μM DTT, and 100 μM NaVa) supplemented with protease and phosphatase inhibitors cocktail (Sigma-Aldrich, St-Louis, USA) and total protein content determined using Bradford reagent. Equal protein content from whole cell lysates was separated on SDS-PAGE. Then, proteins were blotted to nitrocellulose membranes, blocked in 5% BSA in TBST (Tris-buffered saline supplemented by 0.1% Tween 20) for 1 h at RT, and incubated over night at 4 °C in the presence of primary antibodies rabbit anti-SIRPα (Abcam, Cambridge, UK) and mouse anti-α-tubulin (Sigma-Aldrich, St-Louis, USA). Blots were washed with TBST and incubated with respective secondary antibodies goat anti-rabbit and goat anti-mouse conjugated to HRP (Jackson Immuno Research, USA) for 40 min at RT. Proteins were visualized by enhanced chemiluminescence for HRP detection.

### Immunofluorescence microscopy

Nerves were cross-sectioned (15 μm) in a freezing cryostat, sections fixed in 4% paraformaldehyde in PBS for 15 min, washed 3x10 min in PBS containing 0.1% triton (PBST), blocked (10% normal donkey serum, NDS, in PBST) for 1 h at room temperature, and incubated overnight at 4 °C in the presence of primary antibodies as described below. To visualize CD47 and SIRPα, sections were incubated in rat anti-mouse CD47 and SIRPα monoclonal antibody (mAb) (provided by Dr. Oldenborg, Umea, Sweden). To visualize macrophages, sections were incubated in hybridoma supernatants containing rat anti-mouse mAb M1/70 (Developmental Studies Hybridoma Bank, Iowa City, USA) and mAb 5C6 (American Type Culture Collection, Rockville, USA) against αM/CD11b subunit of CR3 (complement receptor-3). To visualize SIRPα in macrophages, sections were double stained with mAbs against αM/CD11b subunit of CR3 (see above) and anti-SIRPα Ab8120 (Abcam, Cambridge, UK). To visualize axons, sections were incubated in anti-neurofilament (Sigma-Aldrich, Saint Louis, USA) (diluted 1:200 in 2% NDS in PBST). The following day, sections were washed 3x10 min in PBS and incubated with the corresponding secondary antibodies: donkey anti- rabbit CY3 1:1000 and FITC-conjugated rabbit anti-rat IgG (Jackson IR laboratories, PA, USA) in 2% NDS for 1 h at room temperature, washed 3X10min in PBS, stained with Hoechst (1:2000) for 2 min, and washed in PBS for 10 min. Finally, sections were air-dried, mounted on coverslips, and sealed with Fluoromount (Sigma-Aldrich, Saint Louis, USA). All steps involving fluorescence were carried out in a slightly darkened room. The immuno-stained slices were examined under a fluorescence microscope.

### Statistical analysis

The following statistical analyses were carried out using GraphPad Prism software: Gaussian distribution, the parametric unpaired *t* test and one- and two-way ANOVA, the nonparametric Mann-Whitney test, and the log-rank Mantel-Cox test. Data that passed the normality test were subjected to parametric statistics and those that were too small for testing for normality were subjected to nonparametric statistics.

## Results

### Macrophages present in Wallerian degeneration express SIRPα protein

**Fig. 1:**
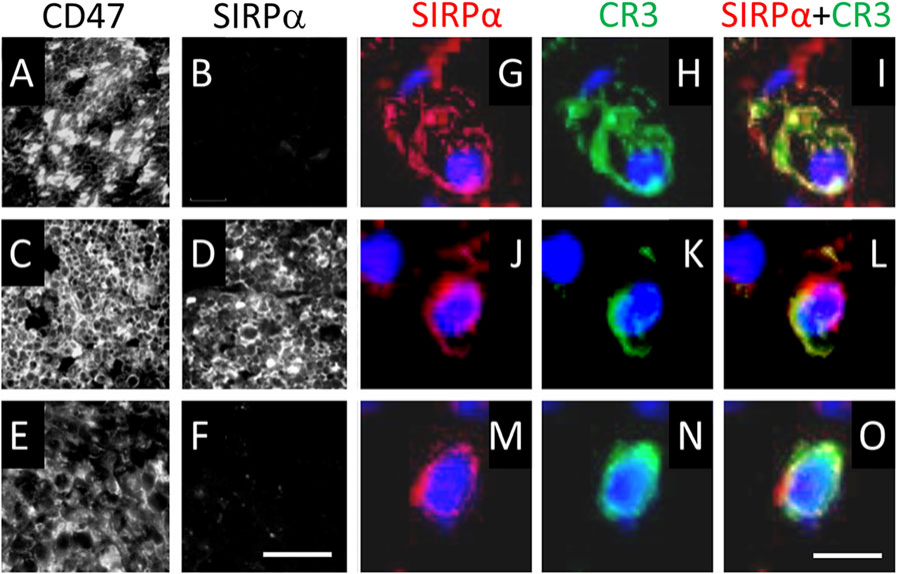
Macrophages present in Wallerian degeneration express SIRPα in wild-type mice. **a**–**f** Cryostat sections in which CD47 (**a**, **c**, **e**) and SIRPα (**b**, **d**, **f**) were visualized by immunofluorescence microscopy using anti-CD47 and anti-SIRPα mAbs. Sections were taken from intact sciatic nerve (**a**, **b**), sciatic nerve undergoing in vivo Wallerian degeneration for 5 days (**c**, **d**), and cultured sciatic nerve explant undergoing in vitro Wallerian degeneration for 5 days (**e**, **f**). All three tissues displayed immunoreactivity to CD47 whereas only sciatic nerve undergoing in vivo Wallerian degeneration displayed immunoreactivity to SIRPα. Bar in **f**: 100 μm, for **a** through **f**. **g**–**o** Three macrophages (**g**–**i**), (**j**–**l**), and (**m**–**o**) from 7 days in vivo Wallerian degenerating nerve segments were visualized by immunofluorescence microscopy using anti-SIRPα (**g**, **j**, **m**; red) and anti-αM/CD11b subunit of CR3 (**h**, **k**, **n**; green) Abs. SIRPα and CR3 combined (**i**, **l**, **o**). Hoechst staining visualized nuclei (blue). The three cells displayed immunoreactivity to both SIRPα and CR3. Bar in **o**: 5 μm, for **g** through **o**.

To examine whether SIRPα impedes the in vivo removal of myelin debris in Wallerian degeneration, it was important to determine which of the two cells that contribute most to the removal of myelin debris, macrophages and Schwann cells [17], express SIRPα protein. We addressed this issue by using immunofluorescence microscopy to visualize SIRPα and its ligand CD47 and the phagocytic receptor CR3 (complement receptor-3; also known as αM/β2 integrin, CD11b/CD18 and MAC-1) that macrophages but not Schwann cells express [17]. Intact nerves, which mostly contain fibroblasts, Schwann cells, and myelin but few macrophages displayed immunoreactivity to CD47 but not SIRPα, indicating that Schwann cells did not express SIRPα (Fig. 1a, b). In contrast, 5 days in vivo Wallerian degenerating nerve segments, which contain numerous macrophages (i.e., cells that express the macrophage specific F4/80 antigen and the phagocytic receptor CR3 [17]) along with fibroblasts, Schwann cells, and myelin, displayed immunoreactivity to both CD47 and SIRPα, suggesting that macrophages expressed SIRPα (Fig. 1c, d). We further confirmed this by showing that cells present in 7 days in vivo Wallerian degenerating nerve segments expressed both CR3 and SIRPα (Fig. 1g–o). However, Schwann cells could express SIRPα as well since Schwann cells undergo a phenotypic change in Wallerian degeneration that makes them resemble macrophages as injury induced Schwann cells to scavenge myelin debris and further express Galectin-3 (formerly named MAC-2) as did macrophages [17]. To address this issue, we used explants of intact nerves that undergo degeneration in culture in the absence of recruited macrophages since Schwann cells displayed the same phenotypic change in nerves that degenerated in culture as in nerves that degenerated in vivo [17]. In vitro degenerating nerve explants (i.e., in vitro Wallerian degeneration) displayed immunoreactivity to CD47 but not SIRPα, indicating that Schwann cells that undergo an injury-induced phenotypic change did not express SIRPα. Our present findings in the nerve tissue as a whole agree with our previous observations that isolated primary Schwann cells and isolated myelin expressed CD47 but not SIRPα whereas primary macrophages expressed both [22]. Taken altogether, macrophages but not Schwann cells express SIRPα in vivo in Wallerian degeneration.

### Deletion of SIRPα protein in macrophages augments the phagocytosis of myelin debris

**Fig. 2:**
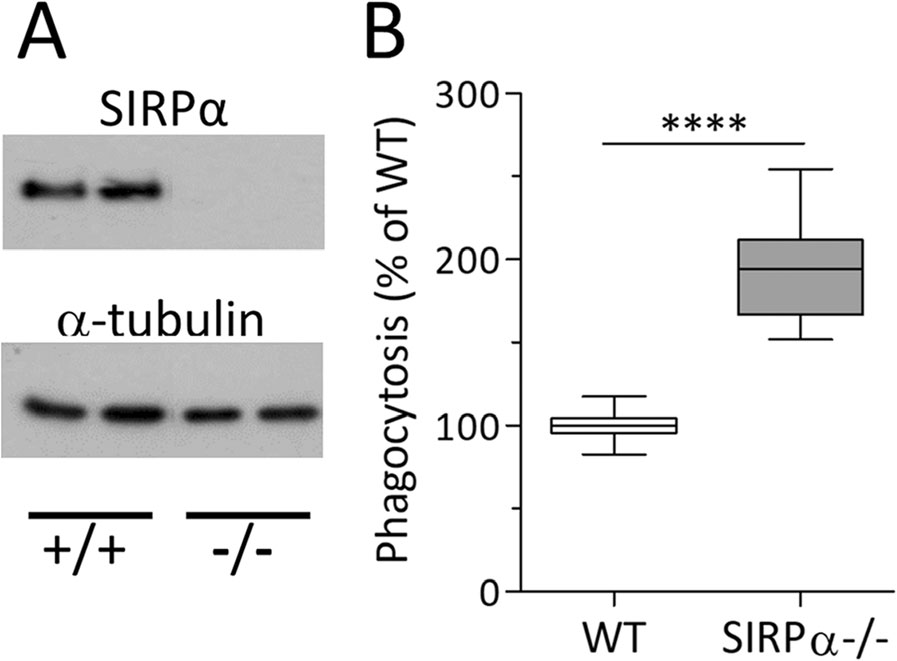
SIRPα inhibits the phagocytosis of myelin debris in cultured BMDM (bone marrow-derived macrophages). **a** BMDM from wild-type (WT; +/+) but not SIRPα−/− (−/−) mice expressed SIRPα protein. Immunoblot of BMDM lysates using anti-SIRPα Ab determined the presence/absence of SIRPα protein (130KD). Immunoblot of same lysates using anti-α-tubulin Ab (55KD) verified equal protein load. **b** BMDM from SIRPα−/− mice (SIRPα−/−) phagocytosed more myelin debris than BMDM from wild-type mice (WT). BMDM plated at low cell density were exposed to myelin debris for 40 min and levels of phagocytosed myelin debris quantified by ELISA. Phagocytosis by SIRPα−/− BMDM is presented as percentage of phagocytosis by wild-type BMDM normalized to 100%. Box and whisker plot of eight experiments is given. The line represents the median, the box outlines the 25 to 75% range, and the whiskers extend to the highest and lowest observations. Double tailed value of significance, ****p < 0.0001, by unpaired *t* test

As we intended to compare between SIRPα−/− and wild-type mice to examine whether SIRPα inhibits the removal of myelin debris in vivo, it was essential to verify that macrophages from SIRPα−/− mice phagocytosed more myelin debris than macrophages from wild-type mice. For this purpose, we used BMDM (bone marrow-derived macrophages) from SIRPα−/− and wild-type mice (Fig. 2). BMDM from SIRPα−/− mice did not express the SIRPα protein and phagocytosed about twofold more myelin debris than BMDM from wild-type mice. We also compared thioglycollate-elicited peritoneal macrophages from the two mice strains and obtained similar results (not shown).

Augmented phagocytosis of myelin debris that genetic deletion of SIRPα produced in BMDM and primary macrophages from SIRPα−/− mice agrees with our previous findings in cultured primary wild-type macrophages and microglia [22, 23]. Function blocking monoclonal antibodies against SIRPα and its ligand CD47, phagocytosis of myelin debris that does not express CD47, and knocking down SIRPα all augmented phagocytosis. Thus, both genetic deletion of SIRPα and genetic-independent prevention of SIRPα activation (i.e., blocking SIRPα-CD47 binding and eliminating CD47 from myelin) augmented phagocytosis, suggesting that both increased the phagocytic capacity of macrophages by eliminating SIRPα-dependent inhibition of phagocytosis and, further, that genetic deletion of SIRPα did not most likely induce another mechanism that augmented phagocytosis. Therefore, SIRPα−/− mice are a suitable model for studying the role of SIRPα in removing myelin debris in vivo.

### The in vivo removal of myelin debris in Wallerian degeneration is faster in SIRPα−/− mice compared with wild-type mice

**Fig. 3:**
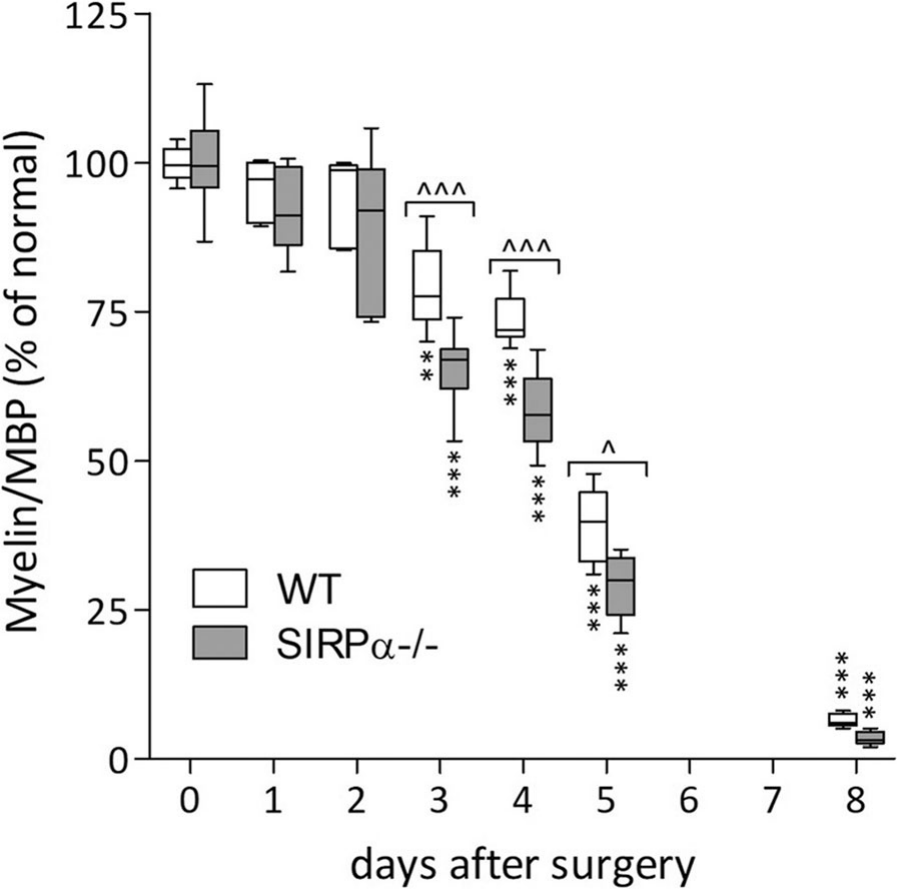
The in vivo removal of myelin debris is faster in SIRPα−/− mice than in wild-type mice. Sciatic nerve segments undergoing Wallerian degeneration were removed from wild-type (WT) and SIRPα−/− mice at the indicated days after surgery, immediately lysed and protein content in lysates quantified. Levels of myelin-specific MBP (Myelin/MBP) in lysate samples of equal protein content were quantified using ELISA. Levels of Myelin/MBP in Wallerian degenerating nerve segments are presented as percentage of levels in intact nerves (time 0) normalized to 100%. Box and whisker plot of Myelin/MBP levels in five to ten different nerves is given. The line represents the median, the box outlines the 25 to 75% range, and whiskers extend to the highest and lowest observations. Significance of difference of WT mice from SIRPα−/− mice at the indicated days after surgery, **^***p* < 0.05 and **^^^***p* < 0.001, by two-way ANOVA and the Bonferroni multiple comparisons posttest. Significance of difference between levels of Myelin/MBP in intact nerves (day 0) and those at the indicated days after surgery, **p < 0.001 and ***p < 0.0001, by one-way ANOVA and the Dunnett posttest calculated for each mice strain separately

Previously, we analyzed the time course of myelin debris removal in Wallerian degeneration by determining the reduction in nerve-tissue content of the myelin-specific proteins MBP and P0 in nerve segments located distal to but not including the lesion sites [33]. We used the same approach to study the time course of myelin debris removal in wild-type and SIRPα−/− mice after cutting the sciatic nerve in the absence of axon regeneration (Fig. 3). Intact nerves from the two mice strains displayed similar MBP content, indicating similar myelin content. Compared with intact nerves, MBP content decreased significantly in the degenerating nerves as of day 3 after surgery in the two mice strains, in agreement with our previous findings [33]. However, MBP content decreased significantly more in SIRPα−/− mice compared with wild-type mice on days 3, 4, and 5 after surgery, suggesting faster removal of myelin debris in SIRPα−/− mice due to the increased phagocytic capacity of SIRPα−/− macrophages present in Wallerian degeneration (Fig. 2). We reached this understanding based on the findings that between the two cells that most scavenge myelin debris, macrophages, and not Schwann cells express SIRPα in wild-type mice (Fig. 1 and [22]), and so, deletion of SIRPα could only have taken place in macrophages, resulting in their increased phagocytic capacity. Thus, in wild-type mice, SIRPα inhibits the phagocytosis of myelin debris in macrophages during in vivo Wallerian degeneration.

### Sensory and motor functions recover faster in SIRPα−/− mice compared with wild-type mice

For studying how Wallerian degeneration affects the growth/regeneration of severed axons and thereby the recovery of functions, it was advantageous to follow the regeneration of as many axons as possible. To achieve this goal, we inflicted freeze-crush injuries to nerves. We chose this type of injury since it ensured the disruption of all axons, further preserved the continuity of the nerve connective tissue, and so enabled a large proportion of severed axons to cross the lesion site and then grow/regenerate throughout the distally located Wallerian degenerating nerve segment.

**Fig. 4:**
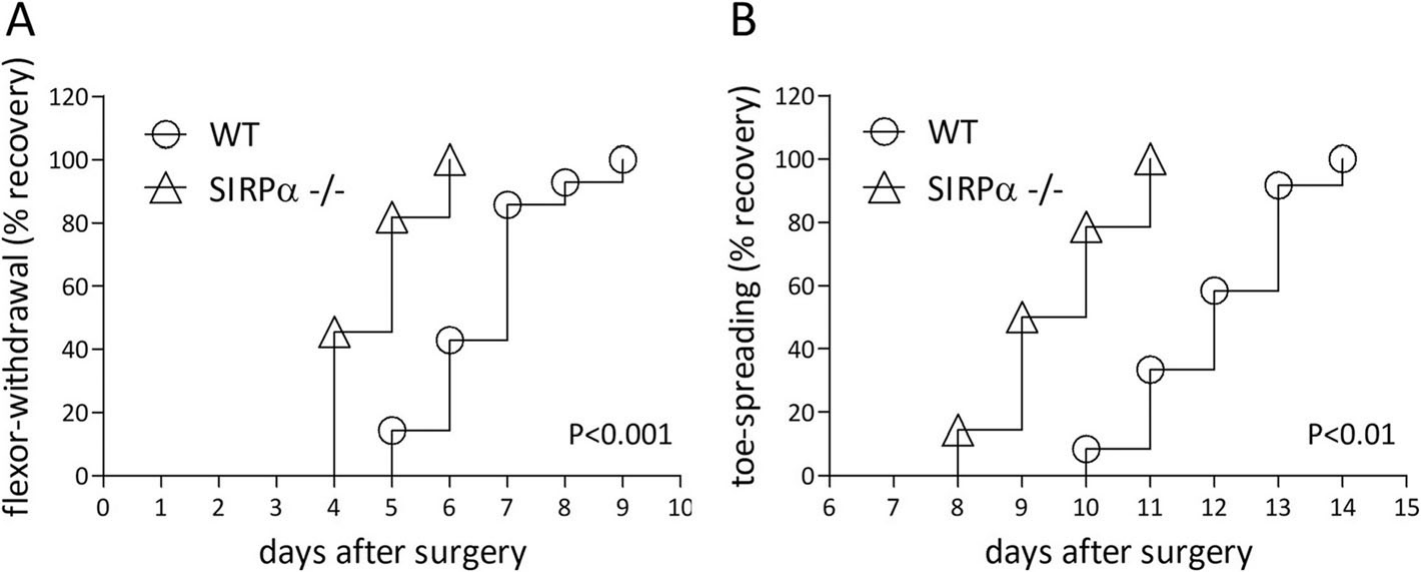
Sensory and motor functions recover faster in SIRPα−/− mice than in wild-type mice. The **a** flexor-withdrawal reflex and **b** toe-spreading reflex recovery curves display the cumulative percentage of mice that regained (**a**) sensory and (**b**) motor functions at each of the indicated days after surgery. **a** Sensory function was tested in 14 wild-type (WT) and 11 SIRPα−/− mice. Significance of difference of WT mice from SIRPα−/− mice, *p* < 0.001, by the log-rank Mantel-Cox test. **b** Motor function was tested in 12 wild-type (WT) and 11 SIRPα−/− mice using the toe-spreading reflex. Significance of difference of wild-type mice from SIRPα−/− mice, *p* < 0.01, by the log-rank Mantel-Cox test.

To test the recovery of sensory function, we used the flexor-withdrawal reflex, hind limb withdrawal in response to gently touching the paw. The saphenous and the sciatic nerves provide sensory innervation to the hind limb paw and the sciatic nerve further supplies motor innervation to hind limb muscles. We freeze-crushed saphenous nerves at an average distance of 14 mm from paws, which enables severed sensory axons to regenerate. At the same time and same limb, we resected a segment of the sciatic nerve at mid-thigh level to prevent axon regeneration but spare hip joint flexion and thereby limb withdrawal. Thus, reflex recovery depended solely on successful regeneration of and skin reinnervation by the regenerating saphenous nerve sensory axons. We operated on and then tested wild-type and SIRPα−/− mice side by side at 1-day intervals after surgery (Fig. 4a). The reflex disappeared for at least 3 days after surgery, confirming successful sensory denervation of paws. In SIRPα−/− mice, the reflex returned in 45% of mice on day 4, median recovery was on day 5, and all mice had regained the reflex by day 6 after surgery. In wild-type mice, the reflex returned in 14% of mice on day 5, median recovery was on day 7, and all mice had regained the reflex by day 9 after surgery. Thus, SIRPα−/− mice regained sensory function faster than wild-type mice. The median recovery times, 5 days in SIRPα−/− mice and 7 days in wild-type mice, indicate that deletion of SIRPα speeded up the recovery of sensory function about 1.4-fold.

To test the recovery of motor function, we used the toe-spreading reflex, spreading of the hind limb toes in response to lifting mice by their tail. We freeze-crushed sciatic nerves at an average distance of 20 mm from hind limb paws, which enables severed motor axons to regenerate and reinnervate abductor muscles of toes on which the recovery of the reflex depended. We operated on and then tested the same wild-type and SIRPα−/− mice side by side at 1-day intervals after surgery (Fig. 4b). The reflex disappeared for at least 7 days after surgery, confirming successful denervation of muscles. In SIRPα−/− mice, the reflex returned in 14% of mice on day 8, median recovery was at 9.5 days, and all mice had regained the reflex by day 11 after surgery. In wild-type mice, the reflex returned in 8% of mice on day 10, median recovery was on day 12, and all mice had regained the reflex by day 14 after surgery. Thus, SIRPα−/− mice regained motor function faster than wild-type mice. The median recovery times, 9.5 days in SIRPα−/− mice and 12 days in wild-type mice, indicate that deletion of SIRPα speeded up the recovery of motor function about 1.26-fold.

### Severed axons grow faster through Wallerian degeneration in SIRPα−/− mice compared with wild-type mice

**Fig. 5:**
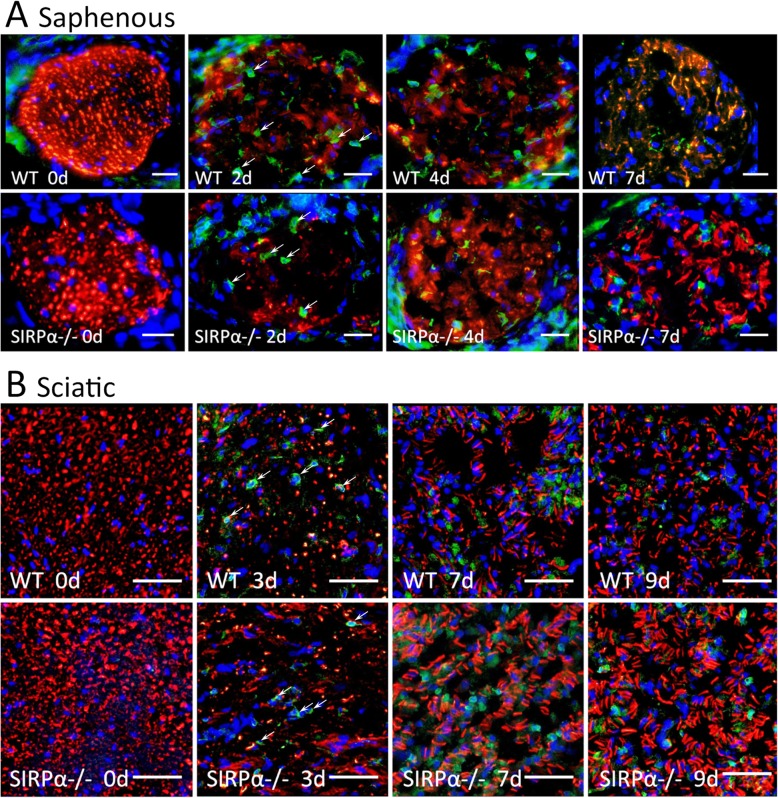
Severed axons regenerate faster in SIRPα−/− mice than in wild-type mice. Axons and macrophages were visualized in intact and Wallerian degenerating (**a**) saphenous and (**b**) sciatic nerves by immunofluorescence microscopy using Abs against neurofilaments (NF; red) for labeling axons and mAbs against the αM/CD11b subunit of CR3 (green) for labeling macrophages. The overlay of NF/red over CR3/green is yellow. Hoechst staining visualized nuclei (blue). **a** Intact (0d) and freeze-crushed saphenous nerves were sampled at distances ranging from 8 to 10 mm distal to lesion sites 2, 4, and 7 days (2d, 4d, and 7d) after surgery. **b** Intact (0d) and freeze-crushed sciatic nerves were sampled at distances ranging from 8 to 10 mm distal to lesion sites 3, 7, and 9 days (3d, 7d, and 9d) after surgery. Initially, on days 2 and 3 after surgery, NF immunoreactivity decreased in wild-type (WT) and SIRPα−/− mice. Then, at the indicated days thereafter, NF immunoreactivity increased more in SIRPα−/− mice than in wild-type mice. CR3 immunoreactivity that was hardly detected in intact nerves (0d) increased as of days 2 and day 3 after surgery onwards in saphenous and sciatic nerves of both WT and SIRPα−/− mice; arrows mark some of the CR3 expressing cells. Shown are representative images from four nerves that were sampled at each time point. Bars: 20 μm in **a** and 100 μm in **b**

The earlier recovery of functions in SIRPα−/− mice compared with wild-type mice (Fig. 4) resulted most likely from the faster growth of severed regenerating axons. To test this possibility, we used immunofluorescence microscopy to visualize axons and macrophages in intact and freeze-crushed Wallerian degenerating nerve segments 8 to 10 mm distal to lesion sites that were excluded (Fig. 5). Immunoreactivity to NF (neurofilaments) visualized axons and immunoreactivity to CR3 (complement receptor-3) visualized macrophages (detailed in the next section).

**Fig. 6:**
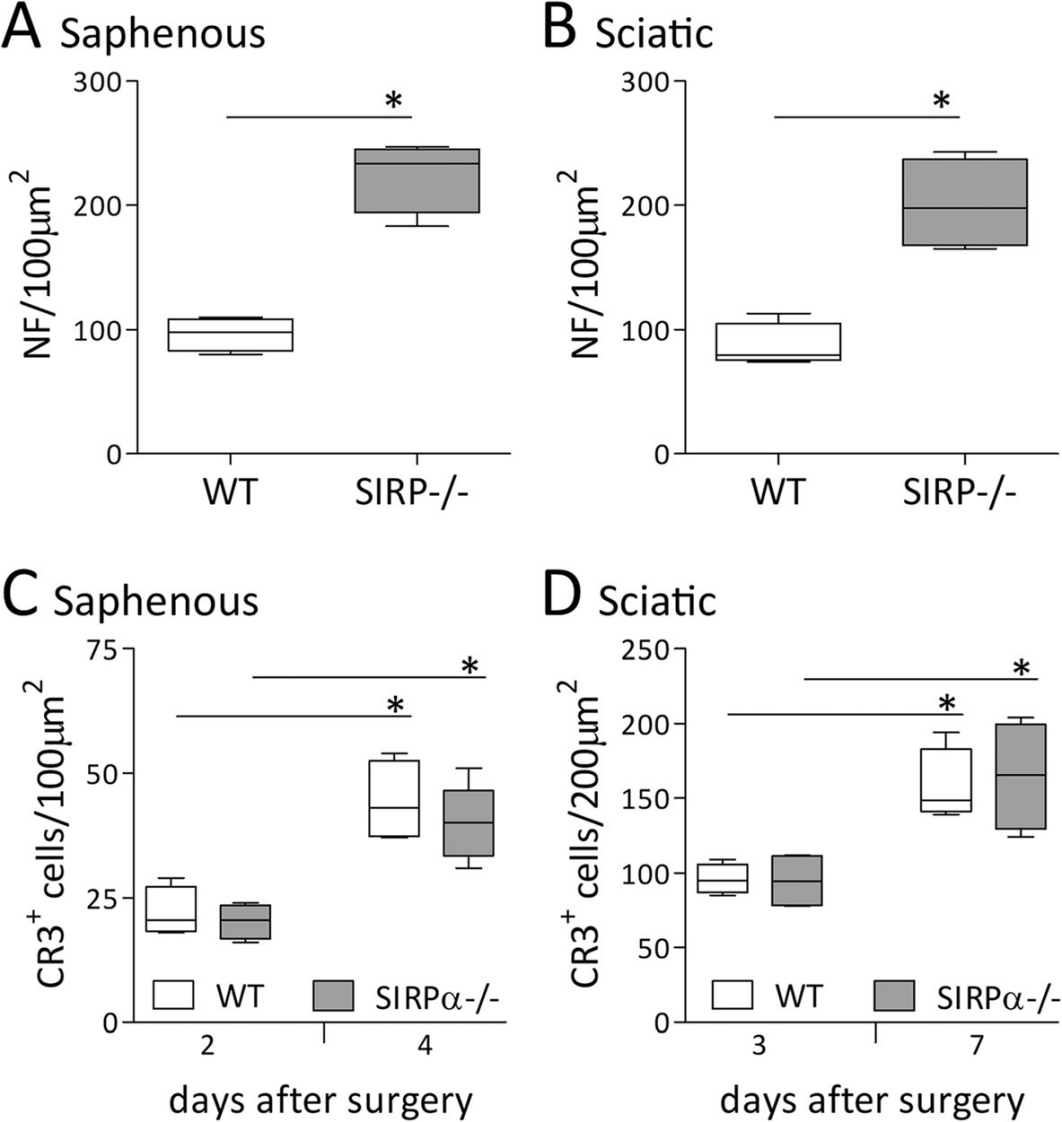
Regenerating axons appear faster in SIRPα−/− than in wild-type mice but the number of CR3 expressing cells present in Wallerian degeneration are comparable in the two mice strains. **a**, **b** Neurofilaments (NF) were counted in cryostat sections from wild-type (WT) and SIRPα−/− mice (Fig. 5) in 100 μm^2^ areas of **a** saphenous nerves 4 days after surgery and **b** sciatic nerves 7 days after surgery. The number of NF images that label axons was about twofold higher in SIRPα−/− than in wild-type mice in the two nerves. Significance of difference of the number of NF images between SIRPα−/− and wild-type mice, *p < 0.05, by Mann-Whitney test. **c**, **d** Cells that express the phagocytic receptor CR3 were counted in cryostat sections from wild-type (WT) and SIRPα−/− mice (Fig. 5) at the indicated days after surgery in **c** 100 μm^2^ area of saphenous nerves and **d** 200 μm^2^ area of sciatic nerves. Green images (CR3-labeled cells) and yellow images (green, CR3-labeled cells overlaying red, NF-labeled axon) were identified as CR3-expressing cells. The number of CR3 expressing cells increased significantly with time after surgery in both sciatic and saphenous nerves but to same levels in the two mice strains. Significance of difference of the number of CR3 expressing cells between days 2 and 4 in saphenous nerves and days 3 and 7 in sciatic nerves from SIRPα−/− and wild-type (WT) mice, *p < 0.05, by Mann-Whitney test. Box and whisker plots of the number of NF and CR3 expressing cells from four different nerves at each indicated day after surgery are given. The line represents the median, the box outlines the 25% to 75% range, and whiskers extend to the highest and lowest observations

Saphenous nerves were injured 14 mm from paws and then sampled 4 to 6 mm from paws on days 2, 4, and 7 after surgery (Fig. 5a). NF immunoreactivity decreased remarkably in both wild-type and SIRPα−/− mice on day 2 after surgery, indicating loss of axons due to rapid degeneration. NF immunoreactivity increased markedly in SIRPα−/− mice but little in wild-type mice on day 4 after surgery, indicating quicker appearance of newly regenerating axons at the sampling site in SIRPα−/− mice compared with wild-type mice. We confirmed this by showing that the number of neurofilaments that mark axons was about twofold higher at the sampling site in SIRPα−/− mice compared with wild-type mice (Fig. 6a). On day 7 after surgery, NF immunoreactivity increased further in both mice strains, indicating the appearance of additional newly regenerating axons at the sampling site. These observations are in good agreement with the loss of sensory function in all mice for the first 3 days after surgery, functional recovery in 45% of SIRPα−/− mice but in none of wild-type mice on day 4 after surgery, and functional recovery in all SIRPα−/− mice and 85% of wild-type mice on day 7 after surgery (Fig. 4a).

Sciatic nerves were injured 20 mm from paws and then sampled 8 to 12 mm from paws on days 3, 7, and 9 days after surgery (Fig. 5b). NF immunoreactivity decreased remarkably in both wild-type and mice SIRPα−/− on day 3 after surgery, indicating loss of axons due to rapid degeneration. NF immunoreactivity increased markedly on days 7 and further on day 9 after surgery but more in SIRPα−/− than in wild-type mice, suggesting quicker appearance of newly regenerating axons in SIRPα−/− compared to wild-type mice. We confirmed this by showing that the number of neurofilaments that mark axons was about twofold higher at the sampling site in SIRPα−/− mice compared with wild-type mice (Fig. 6b). These findings are in good agreement with the loss of motor function in all mice for the first 7 days after surgery and functional recovery in 50% of SIRPα−/− mice but in none of the wild-type mice on day 9 after surgery (Fig. 4b).

### The number of CR3 expressing phagocytes present in Wallerian degeneration in wild-type and in SIRPα−/− mice are comparable

The faster removal of myelin debris in SIRPα−/− mice compared with wild-type mice (Fig. 3) could have resulted not only from the more efficient phagocytic capacity of SIRPα−/− macrophages compared with wild-type macrophages (Fig. 2) but also from differences between the two mice strains with respect to the number of macrophages present in Wallerian degeneration. We addressed this issue by quantifying the number of cells that express CR3 since, as we previously documented; CR3 mediated much of the phagocytosis of myelin debris in macrophages and microglia [18–20]. For this purpose, we sampled the same intact and freeze-crushed nerves in which we studied axon degeneration and regeneration (Fig. 5), thus 8 to 10 mm distal to lesion sites that were excluded. CR3 expressing cells were visualized by detecting the immunoreactivity to CD11b/αM subunit of CR3. Immunoreactivity to CR3 was infrequently observed in intact nerves from the two mice strains, which agrees with the detection of 1.2 macrophages per 100 μm^2^ in intact nerves [34]. The number of CR3 expressing cells increased progressively and to similar levels in the two mice strains from days 2 and 3 after surgery, the earliest that we tested, continuing to days 4 and 7 after surgery, the later post-injury days that we tested (Fig. 6c, d). These observations agree with our previous findings that the number of macrophages (i.e., cells expressing the macrophage specific F4/80 antigen) increased progressively from 2.5 to 7 days after surgery [17, 35–37]. Noteworthy, CR3 expressing cells could be both macrophages and neutrophils [37, 38]. However, most were macrophages since macrophages outnumber neutrophils through the entire period of myelin debris removal (see the “Discussion” section).

## Discussion

This study is the first to reveal an intrinsic normally occurring SIRPα-dependent mechanism that impedes the in vivo removal of myelin debris by inhibiting phagocytosis in macrophages present in Wallerian degeneration, leading to slow axon growth/regeneration through Wallerian degeneration and thereby to delayed recovery of function from nerve injury. We suggest this mechanism based on the following major results and arguments. First, compared with wild-type/SIRPα+/+ mice, deletion of SIRPα increased the phagocytic capacity of macrophages and further accelerated the removal of myelin debris in SIRPα−/− mice. Since macrophages and Schwann cells scavenge most of myelin debris in Wallerian degeneration, and only macrophages express SIRPα in wild-type/SIRPα+/+ mice, deletion of SIRPα could only have taken place in macrophages and not in Schwann cells. Therefore, the accelerated removal of myelin debris in SIRPα−/− mice is most likely due to the increased phagocytic capacity of SIRPα−/− macrophages. Second, compared with wild-type/SIRPα+/+ mice, deletion of SIRPα accelerated axon regeneration through Wallerian degenerating nerve segments and so facilitated the recovery of functions in SIRPα−/− mice. The faster removal of axon growth-inhibitory myelin debris in SIRPα−/− mice accounts most likely for faster axon growth. This is suggested by the findings in wild-type/SIRPα+/+ mice that the slower removal of myelin debris was associated with slower axon growth and delayed recovery, supported by live imaging of myelin debris slowing axon growth [21]. All those findings together suggest that in SIRPα−/− mice, deletion of SIRPα increases the phagocytic capacity of macrophages, leading to faster removal of axon growth-inhibitory myelin debris, and so to accelerated axon growth and facilitated recovery of functions. By contrast, in wild-type/SIRPα+/+ mice, SIRPα impedes the phagocytic capacity of macrophages, leading to protracted removal of myelin debris that slows axon growth, and so prevents severed axons from fully implementing their regenerative potential.

The role that we suggest to assign to SIRPα in the recovery of function from nerve injury requires that genetic deletion of SIRPα increases the phagocytic capacity of SIRPα−/− macrophages by eliminating SIRPα-dependent inhibition of phagocytosis and not by inducing another mechanism. Our previous findings in cultured primary wild-type/SIRPα+/+ macrophages and microglia suggest that this is the case. Function blocking antibodies against SIRPα and its ligand CD47, phagocytosis of myelin debris that does not express CD47, and knocking down SIRPα all augmented phagocytosis [22, 23]. Since both genetic deletion of SIRPα and the genetic-independent prevention of SIRPα activation (i.e., blocking SIRPα-CD47 binding and eliminating CD47 from myelin debris) augmented phagocytosis, the increased phagocytic capacity is most likely due to the elimination of SIRPα-dependent inhibition of phagocytosis and not by another mechanism that genetic deletion of SIRPα may have induced.

Another point of consideration is whether deletion of SIRPα facilitated axon growth and the recovery of function by preventing SIRPα from directly inhibiting axon growth by acting on or in neurons/axons and not indirectly by preventing SIRPα from impeding the removal of axon growth-inhibitory myelin debris, which we suggest. Experimental findings in mice that express SIRPα support our proposition and not the alternative. For example, the deletion of axon growth-inhibitory MAG from myelin in mice that also display exceptionally slow removal of myelin debris advanced the growth of severed axons in the presence of myelin debris [13], and further, axons grew faster through Wallerian degenerating nerve segments from which myelin debris had previously been removed (discussed below under “conditioning injury”).

Neutrophils, which infiltrate the Wallerian degenerating nerve early but transiently after injury, could phagocytose and so contribute to the removal of myelin debris [37, 38]. Two scenarios warrant consideration in this regard. First, neutrophils, as macrophages, express both the phagocytic receptor CR3 and inhibitory SIRPα [39]. Thus, deletion of SIRPα should augment phagocytosis in neutrophils as in macrophages. Second, it is most probable that the overall contribution of macrophages to the phagocytosis of myelin debris is considerably greater than that of neutrophils since the presence of neutrophils in Wallerian degeneration is transient, and further, macrophages outnumber neutrophils exceedingly through the entire period of myelin debris removal. We reached this understanding based on the findings that neutrophils were outnumbered by macrophages 3 to 1 on day 3 after injury, and then, at day 7 after injury, neutrophils were practically absent [38]. In contrast and during the same period, the number of macrophages (i.e., cells expressing the murine macrophage specific F4/80 antigen) increased progressively [17, 35–37]. Thus, in Wallerian degeneration, a sustained presence of numerous macrophages replaces an initial short-lived presence of substantially fewer neutrophils through the entire period of myelin debris removal.

Our proposition that macrophages phagocytosed exceedingly more myelin debris than neutrophils seemingly contradicts the finding that deletion of neutrophils reduced myelin debris removal [38]. This apparent discrepancy can be reconciled if neutrophils contributed to macrophage recruitment in Wallerian degeneration as they do in acute inflammation in other tissues. In acutely inflamed tissues, an initial transient infiltration of short-lived neutrophils was replaced by a sustained presence of macrophages [40, 41]. This transition was governed by the constitutive apoptotic death of the infiltrating neutrophils and the associated shedding of IL6R (interleukin-6 receptor) from their surface, leading to IL6 trans-signaling that inhibited the production of leukocyte chemoattractants and at the same time induced the production of monocyte chemoattractants. In agreement with that, depletion of neutrophils resulted in reduced levels of soluble IL6R and reduced accumulation of macrophages in inflamed tissues, confirming that normally the initial infiltration of neutrophils prompted macrophage recruitment [41]. We previously showed that fibroblasts produce significant levels of IL6 in Wallerian degeneration within hours after nerve injury, and further, macrophages also produce IL6 but at later stages [42]. The early production of IL6 in Wallerian degeneration [42] and the early recruitment of neutrophils to Wallerian degeneration [38] together suggest that neutrophils could have contributed to macrophage recruitment in Wallerian degeneration as they do in acute inflammation [40, 41].

Our findings highlight the fact that fast removal of myelin debris is critical to recovery from nerve injury and, further, that the contribution of macrophages to the process is significant. In agreement with this understanding are the observations that excluding macrophages from Wallerian degeneration reduced the removal of myelin debris and impaired recovery [43–45] and, further, that the slower removal of myelin debris delayed axon regeneration and muscle reinnervation in aged mice compared with young mice. The removal of myelin debris adds to additional innate-immune functions significant to recovery that macrophages carry out. For example, we previously documented that macrophages in Wallerian degeneration were poor producers of the inflammatory cytokines TNFα (tumor necrosis factor-α), IL1β and GM-CSF (granulocyte-macrophage colony-stimulating factor) [27, 46]. In contrast, the same macrophages were major producers the cytokines IL10 and IL6 [33, 42]; IL10 is a major anti-inflammatory cytokine [47] and IL6 has context-dependent anti-inflammatory and inflammatory properties [48, 49] as well as neurotrophic properties [50]. Thus, macrophages in Wallerian degeneration are mostly of the M2 tissue repair phenotype, reviewed and discussed in [2, 3, 51–53]. Interestingly, a transient short-lived infiltration of neutrophils preceded the infiltration of macrophages that then acquired the M2 phenotype at sites of traumatic injury to spinal cords [54].

The rate of myelin debris removal may affect fastest growing axons most and slower growing axons less or not at all. We base this suggestion on the time course of myelin debris removal in wild-type mice shown here and previously [33] and the fact that the rate of axon growth can vary over a wide range; e.g., from 0.28 to 2.16 mm/day [21]. Hence, fastest growing axons may precede debris removal and debris removal may precede slowest growing axons. Consequently, fastest growing axons may confront more residual myelin debris than slower growing axons and slowest growing axons may not confront myelin debris at all.

It is of interest to interpret previous findings by others in light of our current results and understanding. Rat axons that regenerated through Wallerian degenerating nerve segments grew slower after a single injury than after a test injury that followed a previously inflicted “conditioning injury”, e.g., [55]. Sensory axons regenerated 4.02 mm/day after a single injury and 6.76 mm/day after a test injury that followed a conditioning injury inflicted 7 days before [55]. The authors of this study suggested that cell body changes and the environment of the lesioned axons acted in an additive fashion to produce a maximal conditioning lesion phenomenon. Most studies that followed focused on mechanisms within and surrounding the cell bodies of the axotomized neurons to explain faster regeneration after conditioning lesions, reviewed and discussed in [52]. Our study suggests that the removal of growth-inhibiting myelin debris from the path through which axons regenerate accelerates axon growth and recovery. Taking the normal time course of myelin debris removal in Wallerian degeneration that we showed here and previously [33], the 7-day time interval between conditioning and test injuries enabled removing most of the myelin debris before severed axons began growing after the test injury. By contrast, very little of myelin debris was removed by the time regeneration commenced after a single injury. Notably, the conditioning lesion increased the rate rat sensory axons regenerated 1.68-fold [55]. This is comparable to the 1.4-fold increase in the recovery rate of sensory function caused by removing SIRPα-dependent inhibition of myelin debris removal in mice that we report here since accelerated recovery of function is mostly due to sensory axons regenerating faster.

The speed at which severed axons regenerate through Wallerian degenerating nerve segments affects recovery since axon growth support that initially develops in the Wallerian degenerating nerve tissue declines with time. In rat, recovery deteriorated progressively after delaying regeneration by 8 to 24 weeks and GDNF (glial cell line-derived neurotrophic factor) that Schwann cells initially upregulated declined gradually as of 4 weeks after nerve injury [9, 56]. In mice, levels of c-Jun that Schwann cells upregulated early after nerve injury dropped significantly at 10 weeks after injury [57].

A progressive slowdown of axon regeneration through Wallerian degenerating nerve segments occurs in humans both after nerve crush injuries that did not require reconstructive surgery and after more extensive nerve injuries that did. The regeneration of sensory axons slowed from 2.5 mm/day to 0.5 mm/day and the regeneration of motor axons from 2 mm/day to 1 mm/day [6, 7]. The findings in rodents [9, 56, 57] raise the possibility that the progressive slowdown of axon regeneration in humans is due to, at least in part, to the gradual time-dependent decline of axon growth support that Wallerian degeneration provides. If that is the case and axon growth-support declines considerably at 10 weeks after injury in humans, then axons regenerating 2 mm/day may face injured nerve tissue with reduced ability to support axon regeneration at a distance of about 14 cm from an injury site (i.e., about half or less the distance from elbow to wrist in adults). Thus, regenerating axons are likely to cover longer distances before axon growth support deteriorates by speeding up their rate of growth through the Wallerian degenerating nerve segment.

Our findings that SIRPα inhibits myelin debris phagocytosis in macrophages present in Wallerian degeneration that follows nerve crush injury is most likely relevant to all types of nerve injury since Wallerian degeneration follows all of them. Our study further suggests that blocking SIRPα-dependent inhibition of myelin debris leads to faster regeneration and so to facilitated recovery of function. However, this mechanism can only affect severed axons that successfully cross the lesion site and then enter the distal nerve segment. In humans, nerve crush injuries, as we used in this study, favor crossing and entry to the distal nerve segment whereas more extensive nerve injuries (e.g., cut and avulsion) require surgical reconstruction to achieve this goal. Unfortunately, depending on the nature of trauma, surgical reconstruction has its limitation, discussed in [4, 5].

## Conclusion

Our study in mice suggests that blocking SIRPα-dependent inhibition of myelin debris phagocytosis in macrophages present in the Wallerian degeneration leads to faster removal of axon growth-inhibitory myelin debris and so to accelerated axon growth and facilitated recovery of function. SIRPα and its ligand CD47 are expressed in humans and SIRPα-CD47 interaction has been targeted to treat human cancer [58, 59]. Thus, it may be useful to consider targeting SIRPα-CD47 interaction to accelerate the removal of myelin debris in Wallerian degeneration and additional pathologies of the nervous system in which myelin breaks down and impedes healing (e.g., multiple sclerosis).

## Data Availability

Not applicable.

## Abbreviations

BMDM: Bone marrow-derived macrophages
CR3: Complement receptor-3
GDNF: Glial cell line-derived neurotrophic factor
GM-CSF: Granulocyte-macrophage colony-stimulating factor
IL6R: Interleukin-6 receptor
MAG: Myelin-associated glycoprotein
MBP: Myelin basic protein
MCSF: Colony-stimulating factors
NF: Neurofilaments
SIRPα: Signal regulatory protein-α
TNFα: Tumor necrosis factor-α
